# Genome-Wide Identification and Characterization of TCP Family Genes in Pak-Choi [*Brassica campestris* (syn. *Brassica rapa*) ssp. *chinensis* var. *communis*]

**DOI:** 10.3389/fpls.2022.854171

**Published:** 2022-05-09

**Authors:** Feiyi Huang, Churan Shi, Yuhang Zhang, Xilin Hou

**Affiliations:** State Key Laboratory of Crop Genetics & Germplasm Enhancement, Key Laboratory of Biology and Genetic Improvement of Horticultural Crops (East China), Ministry of Agriculture and Rural Affairs of the P. R. China, Engineering Research Center of Germplasm Enhancement and Utilization of Horticultural Crops, Ministry of Education of the P. R. China, Nanjing Suman Plasma Engineering Research Institute, Nanjing Agricultural University, Nanjing, China

**Keywords:** abiotic stress, expression analysis, subcellular localization, TCP, Pak-choi

## Abstract

The TEOSINTE BRANCHED1/CYCLOIDEA/PROLIFERATING CELL FACTOR (TCP) gene family, a kind of plant specific transcription factor, is essential for stress response, cell growth, and cell proliferation. However, the characterization of TCP family is still not clear in Pak-choi [*Brassica campestris* (syn. *Brassica rapa*) ssp. *chinensis* var. *communis*]. In this study, genome-wide analysis of TCP gene family was performed and 26 *TCP* genes were identified in Pak-choi. Phylogenetic analysis demonstrated that the 26 BcTCPs were divided into two classes: Class I and Class II. Class II was further classified into two subclasses, CIN and CYC/TB1. The qPCR results suggested that most *BcTCPs* respond to abiotic stresses. The expressions of *BcTCP3*, *BcTCP12*, *BcTCP21*, and *BcTCP22* were significantly changed under ABA and cold treatment. *BcTCP3* and *BcTCP12* were also up-regulated under osmotic treatment. Subcellular localization showed that BcTCP3 and BcTCP21 were located in the nucleus. Our results will facilitate revealing the functions and regulatory mechanisms of *BcTCPs*.

## Introduction

The TCP gene family is a class of plant-specific transcription factors and plays important roles in plant growth and development. TCP was named from four genes members: *TEOSINTE BRANCHED 1* (*TB1*) from maize (*Zea mays*) (Doebley et al., 1995), *CYCLOIDEA* (*CYC*) from snapdragon (*Antirrhinum majus*) (Luo et al., 1996), and the *PROLIFERATING CELL FACTORS 1* (*PCF1*) and *PCF2* from rice (*Oryza sativa*) (Kosugi and Ohashi, 1997). *TB1* is a key factor to control apical dominance in maize (Doebley et al., 1997). *CYC* involves in the regulation of floral asymmetry in snapdragons. PCF1 and PCF2 bind to the promoter of *PROLIFERATING CELL NUCLEAR ANTIGEN* (*PCNA*) gene in rice, which involves in DNA replication and repair, maintenance of chromatin structure, chromosome segregation, and cell-cycle progression (Luo et al., 1996; Kosugi and Ohashi, 1997). TCP proteins contain a 59-amino-acid domain with a bHLH motif that involved in DNA binding and protein-protein interaction (Martin-Trillo and Cubas, 2010). According to the conserved domain, TCP proteins can be divided into two classes: Class I (TCP-P Class) and Class II (TCP-C Class) (Kosugi and Ohashi, 2002; Navaud et al., 2007). Class II can be further divided into two subclades: CIN and CYC/TB1 (Martin-Trillo and Cubas, 2010). Class I includes *PCF1* and *PCF2* in rice, *TCP8*, *TCP9*, *TCP14*, *TCP20*, etc. in *Arabidopsis*. Class II contains *TB1* in maize, *TCP1*, *TCP2*, *TCP4*, *TCP10*, *BCR1*, *BCR2*, etc. in *Arabidopsis*.

Abiotic stress has an important effect on plant growth and productivity. Cold stress slows down the metabolic processes of plants, inhibits auxin transport and root elongation (Shibasaki et al., 2009). Salt stress leads to the accumulation of toxic ions in cells, which influences plant absorption of nutrients and water (Boudsocq and Laurière, 2005). Plants are subjected to dehydration under low temperature, high salinity, and drought environmental conditions, of which cause osmotic stress, inducing Abscisic acid (ABA) biosynthesis. ABA functions in multiple stresses, regulating genes related to dehydration and cold stress and controlling osmotic stress tolerance (Yamaguchi-Shinozaki and Shinozaki, 2006; Fujita et al., 2011). Moreover, ABA can reduce water loss by governing stomatal closure (Kim et al., 2010). The growth and development of plants will be severely affected when plants suffer from abiotic stresses. Hence, the study of response mechanisms during abiotic stresses is vital for species. At present, it is reported that many genes of the TCP family are related to various stresses. TCP20 responds to nitrate availability by interacting with NIN-like protein 6 (NLP6) and NLP9, controlling plants root growth (Guan et al., 2017). *OsTCP19* is up-regulated under drought, salt, and cold stress, indicating *OsTCP19* may involve in the stress tolerance (Mukhopadhyay and Tyagi, 2015). In *Cicer arietinum*, five TCP genes (*CaTCP3*/*13*/*15*/*20*/*21*), which contained the MYB *cis*-elements, were strongly induced under drought conditions, and similar results were found in other legumes (Ling et al., 2020). *Oryza sativa miR319* (*Osa-miR319*), an upstream gene of *OsPCF5*, *OsPCF6*, *OsPCF8*, and *OsTCP21*, is decreased in the cold condition in rice. Meanwhile, overexpressing *Osa-miR319* results in down-regulation of its downstream target genes and enhances cold resistance, impling that TCP21 could involve in cold stress (Yang et al., 2013; Wang et al., 2014). In creeping bentgrass (*Agrostis stolonifera*), overexpressing *Osa-miR319* also reduces the expression of its target genes (*AsPCF5*, *AsPCF6*, *AsPCF8*, and *AsTCP14*), enhancing salt and drought tolerance (Zhou et al., 2013). GmTCP8 interacts with GmPYL10 and involves in the ABA signal pathway in soybean (Feng et al., 2018). *ZmTCP42* is associated with ABA and drought stress, which plays an active role in drought tolerance (Ding et al., 2019). AtTCP14 represses *ABA1* (*ABA DEFICIENT 1*) and other ABA-related stress genes in *Arabidopsis* seeds (Rueda-Romero et al., 2012).

Until now, the study of the TCP family has been more and more comprehensive, which have been identified in many species including *Arabidopsis thaliana* (Li, 2015), rice (*Oryza sativa*) (Yao et al., 2007), turnips (*Brassica rapa* ssp. *rapa*) (Du et al., 2017), poplar (*Populus trichocarpa*) (Ma et al., 2016), tomato (*Solanum lycopersicum*) (Parapunova et al., 2014), Switchgrass (*Panicum virgatum* L.) (Huo et al., 2019), *Brassica rapa* and *Brassica oleracea* (Liu et al., 2019). Pak-choi [*Brassica campestris* (syn. *Brassica rapa*) ssp. *chinensis* var. *communis*] has gradually become worldwide vegetable crops. However, Pak-choi *TCP* genes and their regulatory mechanisms are still not clear.

In this study, genome-wide analysis of TCP family genes in Pak-choi was performed. 26 *BcTCP* genes were identified in Pak-choi. Phylogenetic relationship, subcellular localization, and conserved motifs were analyzed. QPCR was employed to illustrate the expression patterns of *BcTCPs* under salt, osmotic, cold, and ABA treatment. The results showed that *BcTCPs* may involve in multiple abiotic stresses. Our findings provide a theoretical basis for further research on the potential functions and regulatory mechanisms of *BcTCP* genes.

## Materials and Methods

### Cloning and Identification of *BcTCP* Gene Family Members

Total RNA was extracted from Pak-choi using the RNAeasy Mini Kit (Tiangen, Beijing, China). The CDSs of the *BcTCPs* were amplified with gene specific primers based on the sequences of *AtTCPs* by homology cloning (Huang et al., 2019 and Supplementary Table 1). The PCR products were cloned into the pMD18-T vector before sequencing. Then, the obtained plasmids were sequenced by TSINGKE (Nanjing, China). All putative BcTCP proteins sequences were analyzed using the Pfam database^1^. Additionally, protein molecular weight, isoelectric point, and amino acid length of BcTCPs were computed by the ExPASy ProtParam tool^2^.

### Phylogenetic Tree and *Cis*-Acting Elements Analysis

24 *AtTCP* genes were retrieved from TAIR^3^. Multiple sequence alignments of putative BcTCPs and AtTCPs were performed using Clustal X 2.1. Phylogenetic tree was completed by the Maximum Likelihood (ML) method using MEGA 7.0 software. The bootstrap values were performed with 1,000 replications. PlantCARE^4^ was used to analyze *cis*-acting elements in promoters of each *BcTCP* gene. After classifying and counting the elements on each promoter (Supplementary Table 2), the heat map was made by TBtools.

### Conserved Domain Analysis

The conserved motifs were analyzed using MEME program^5^ with the default settings except the maximum width was set to 200, and the minimum and maximum numbers of motifs were defifined as 2 and 10, respectively.

### Plant Materials and Growth Conditions

The Pak-choi cultivar ‘49caixin’ was selected in our experiment, which has a short life cycle and may flower within two months after sowing under long-day conditions (Tian et al., 2004). Plants were cultivated in pots with medium (Soil matrix and vermiculite, 1:1). All seedlings were grown in a controlled artificial climatic chamber under the same conditions (16 h light at 22°C/8 h dark at 18°C, 60-70% relative humidity). One-month-old seedlings were treated with 100 μM ABA, 250 mM NaCl and 20% PEG6000, respectively. For cold treatment, one-month-old seedlings were transferred to 4°C. The leaves were collected under each stress treatment in a continuous time course (0, 10, 20, 30, 40, 50 min, 1, 2, 3, 4, 8 h) and stored at −70°C immediately for RNA extraction.

### Expression Analysis of *BcTCP* Genes by qPCR

Following the manufacturer’s instructions, total RNA was isolated from leaves using the RNA easy mini kit (Tiangen, Beijing, China). Then first-strand cDNA was synthesized with a RevertAid First Strand cDNA Synthesis Kit (Thermo, Shanghai, China). Primers for qPCR were designed using Primer 5.0 (Supplementary Table 1). QPCR was performed using the SYBR^®^ Premix Ex Taq kit (Takara, Dalian, China). *BcActin* was used as an internal reference gene. Results were calculated by the 2^–ΔΔCT^ method (Livak and Schmittgen, 2001).

### Subcellular Localization Analysis

WOLF PSORT^6^ was used to predict the subcellular localization of the putative BcTCP proteins. To further confirm their subcellular localization, the protein-coding regions of *BcTCP3* and *21* without the termination codon were amplified and then cloned into pCambia 1,302 vector in fusion with GFP at C-terminal end (*35S: GFP*), generating the fusion constructs. The *35S: BcTCP3-GFP* and *35S: BcTCP21-GFP* were generated. Each plasmid was injected into the tobacco leaves by *Agrobacterium tumefaciens*-mediated transient transformation (strain GV3101). The tobacco was incubated for 12 h under darkness. 2-3 days after injection, the fluorescence of GFP was observed and photographed by confocal microscopy (Leica, TCS SP2, Wetzlar, Germany). DAPI (nucleus specific dye) was used as nuclei dye in the experiment.

## Results

### Identification of *TCP*s in Pak-Choi

A total of 26 *TCP* genes in Pak-choi were obtained by homology cloning based on the *Arabidopsis thaliana TCP* genes. These 26 genes were named *BcTCP1*∼*BcTCP26*. The CDS length of the 26 *BcTCP*s was ranged from 651 to 1,395 bp. The physical and chemical properties were further analyzed using the ExPASy ProtParam tool. The molecular weight of 26 putative BcTCP proteins varied from 23.12 to 56.74 kDa. The isoelectric point was ranged from 5.49 to 9.99, and the amino acid length was ranged from 216 to 520 aa (Supplementary Table 3).

### Phylogenetic Analysis

**FIGURE 1:**
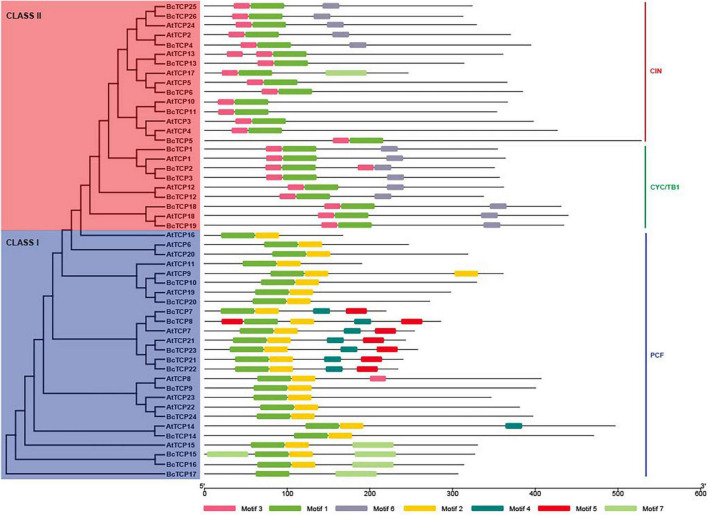
The phylogenetic tree and conserved motifs of BcTCP and AtTCP proteins. Each motif represented with a specific color was shown.

An unrooted phylogenetic tree was constructed with the Maximum Likelihood (ML) method (Figure 1). 26 *BcTCPs* were divided into two classes, Class I and Class II. The Class II *TCP*s were further categorized into two subgroups, CIN and CYC/TB1. PCF Class (Class I) contained 13 genes, CYC/TB1 Class contained 7 genes, CIN only contained 6 genes. Sequence analysis showed that *AtTCP3*, *6*, *11*, *13*, *16*, *17*, *20*, *23* had no ortholog in Pak-choi. *AtTCP1*, *7*, *15*, *18*, *21*, and *24* had more than one ortholog in the Pak-choi.

### Conserved Domain Analysis

The conserved motifs of BcTCP proteins were analyzed using MEME. A total of 7 motifs were found in BcTCPs. The motifs were more similar in the same class. Furthermore, similar conserved motifs arrangements can be observed in the same clade. Motif 1 was conserved in all BcTCPs. Motif 3 and motif 6 only existed in Class II except for BcTCP5, 6, 11, and 13 (Figure 1), suggesting that these BcTCPs may have similar functions. However, proteins among different groups shared diverse motifs. Motif 2 is presented in Class I (PCF) except for BcTCP20. Motif 7 was only existed in BcTCP15, 16, and 17. Motif 4 and motif 5 both existed in BcTCP7, BcTCP8, BcTCP21, BcTCP22, and BcTCP23. These results indicated that TCP genes may perform different functions in Pak-choi.

### Analysis of *Cis*-Acting Elements in *BcTCPs* Promoters

**FIGURE 2:**
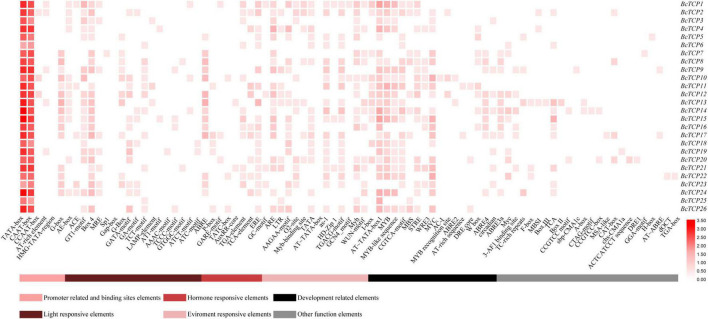
*Cis*-acting elements on promoters of *BcTCP* genes. The color bar showed the number of *cis*-acting elements.

*Cis*-acting elements existed in gene promoters can affect gene expressions and functions. In this study, we found that *BcTCPs* promoters contained not only basic core elements, like TATA-box and CAAT-box, but also a variety of *cis*-acting elements, such as light response elements, hormone response elements, environment response elements, development response elements, and other functional elements (Figure 2). All Class II *BcTCPs* promoters contained G-box elements and ABRE elements except for *BcTCP20* and *BcTCP21*. In Class I *BcTCPs*, the promoters of *BcTCP2*, *3*, *11*, *12*, *13*, *25*, and *26* all contained the low temperature response element (LTR). AT∼TATA box1 was identified in most all *BcTCP* promoters except *BcTCP6* and *BcTCP23*. The promoters of *BcTCP2*, *3*, *4*, *10*, *11*, *12*, *14*, *15*, *20*, *21*, *22*, *26* contained MYB, MYC, and MYB-like sequence elements at the same time. As a result, we hypothesized that these *BcTCP* genes may be involved in stress (Abe et al., 2003; Xu et al., 2021).

### Expression Analysis of *BcTCP* Genes Under Multiple Abiotic Stresses

**FIGURE 3:**
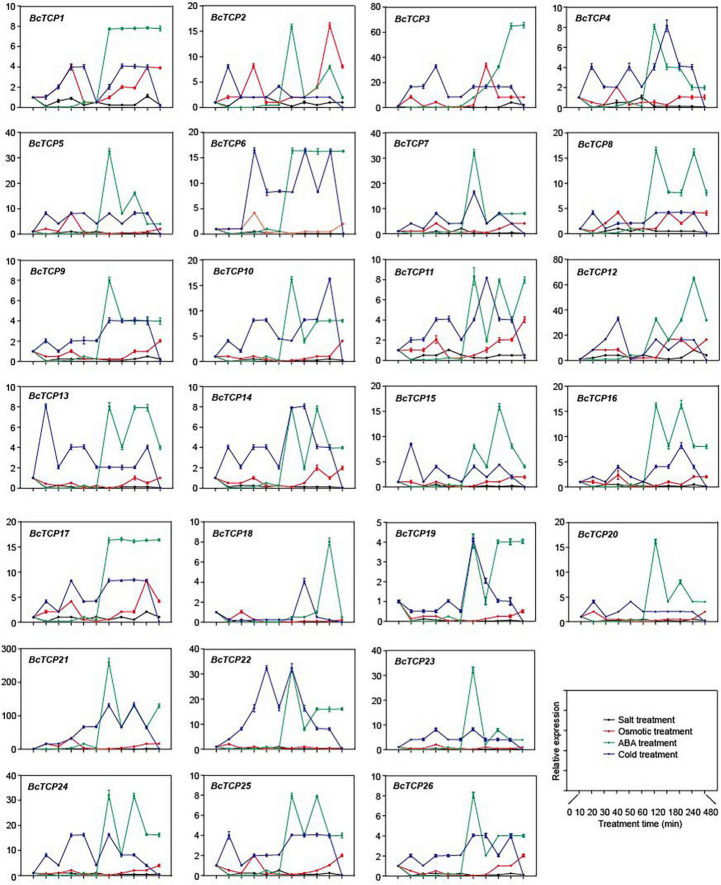
Expression of *BcTCP* genes under different stress treatments. Data shown were means ± SE of three independent experiments.

To investigate the functions of BcTCPs, qPCR was performed to detect the expressions of 26 *BcTCPs* under four treatments (salt, osmotic, cold and ABA) (Figure 3). The results showed that the expressions of most *BcTCPs*, except for *BcTCP12*, were not significantly changed under salt treatment. *BcTCP2*, *3*, *5*, *6*, *7*, *8*, *10*, *12*, *15*, *16*, *17*, *20*, *21*, *22*, *23*, and *24* all up-regulated after 1 h of the ABA treatment. The expression levels of *BcTCP2*, *5*, *6*, *7*, *8*, *10*, *17*, *20*, *21*, *22*, *23*, and *24* reached the maximum at 2 h, *BcTCP15* and *16* had the highest expression at 3h, the largest amount of *BcTCP12* expressed in 4 h, and the largest amount of *BcTCP3* expression quantity in 4 h. Under cold treatment, *BcTCP3*, *6*, *7*, *10*, *12*, *21*, *22*, and *24* showed a significant increase, indicating that these genes, especially *BcTCP21*, may involve in cold stress. Meanwhile, *BcTCP2*, *3*, and *12* were notably up-regulated under osmotic treatment. *BcTCP2* reached its peak at 4 h, and *BcTCP3* and *12* reached their peak at 2 h. These suggested that most *BcTCP* genes were involved in abiotic stresses.

### Subcellular Localization

**FIGURE 4:**
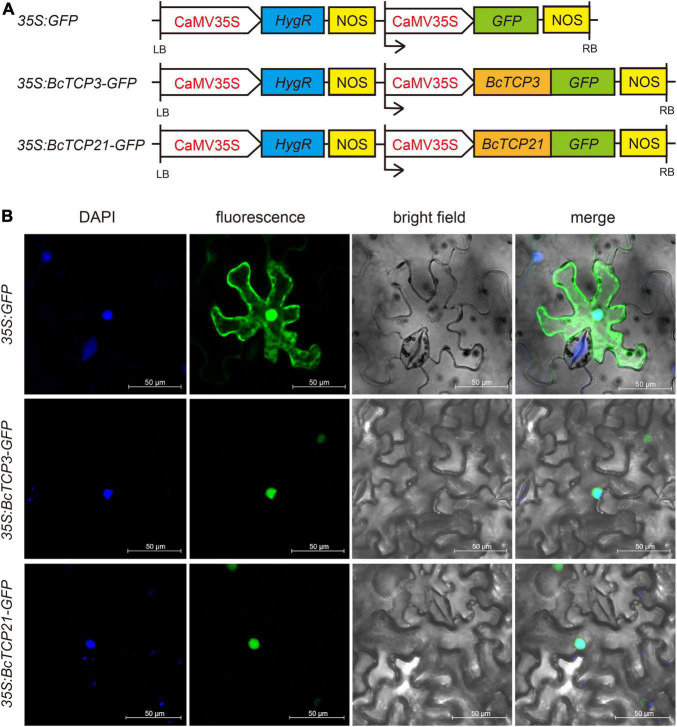
Subcellular localization of BcTCP3 protein and BcTCP21 protein: **(A)** *35S: GFP*, *35S: BcTCP3*, and 35S: BcTCP21 construct. **(B)** Transient expression of 35S:GFP, 35S: 35S: BcTCP3-GFP, and 35S: BcTCP21-GFP fusion protein in tobacco leaves (Scale bars = 50 μm).

WoLF PSORT predicted that BcTCP proteins were localized in the nucleus (Supplementary Table 3). To test this, *35S: GFP*, *35S: BcTCP3-GFP*, and *35S: BcTCP21-GFP* constructs were transiently overexpressed in tobacco leaves (Figure 4A). As seen in Figure 4B, *35S: GFP* was localized in the nucleus and cytoplasm. The GFP signals emitted by BcTCP3 -GFP and BcTCP21 -GFP fusion protein were detected in the nucleus and overlapped with the DAPI staining. This results indicated that BcTCP3 and BcTCP21 were nuclear-localized proteins and may function as transcription factors.

## Discussion

TCP gene family involves in multiple biological processes, including the circadian clock, abiotic stresses, leaf sizes and shapes, flower development and flowering (Takeda et al., 2006; Pruneda-Paz et al., 2009; Mimida et al., 2011; Niwa et al., 2013; Liu et al., 2017; Feng et al., 2018). The roles of TCP genes under abiotic stresses have been reported in some model plants, such as rice, *Zea mays*, *Arabidopsis thaliana*, and soybean (Rueda-Romero et al., 2012; Mukhopadhyay and Tyagi, 2015; Feng et al., 2018; Ding et al., 2019). However, virtually less systematic and comprehensive information of the TCP gene family in Pak-choi was reported, which is a nutritious and economically important vegetable crop and widely cultivated in Asia. Unlike *A. thaliana*, *Brassicas* crops not only underwent this complex evolutionary history, but also the whole-genome triplication (WGT) event between 13 and 17 million years ago (MYA). These events were essential for evolution and resulted in differences among species (Wang et al., 2011). *A. thaliana* has been identified 24 *TCP* genes (Yao et al., 2007). In this study, 26 *BcTCP* genes were isolated and studied in Pak-choi. These *BcTCP*s were divided into Class I and Class II, Class II was then divided into CIN and CYC/TB1, which was consistent with the previously described in *Arabidopsis*, rice, tomato, and strawberry (Yao et al., 2007; Parapunova et al., 2014; Wei et al., 2016). The phylogenetic analysis found that 16 *AtTCP*s had orthologs in Pak-choi. Among them, *AtTCP1*, *7*, *15*, *18*, *21*, and *24* had more than one ortholog. This indicated that BcTCP family genes and AtTCP family genes may have some similar functions. According to conserved domain, we found that the similarity of BcTCPs in the same class is higher than that of different classes of BcTCPs. For example, CYB/TB1 members all contained motif 1, motif 3, and motif 6. Analysis of promoter regions showed that some *BcTCPs* promoters contained MBS *cis*-regulatory elements, MYB *cis*-regulatory elements, ABRE elements, and G-box, suggesting that the *BcTCP* genes might play significant roles in stress responses (Abe et al., 2003; Xu et al., 2021).

QPCR analysis demonstrated that some *BcTCP*s were involved in multiple abiotic stress. For instance, *BcTCP3*, *12*, *21*, *22*, and *24* all responded to cold and ABA treatment. *BcTCP3* had no response to salt stress but had relatively obvious responses under cold and ABA treatments. *BcTCP12* responded to salt, osmotic, ABA, and cold treatments. Under salt treatment, the expression of *BcTCP12* was significantly activated, indicating that *BcTCP12* may enhance salt tolerance, which is same as previous studies in Oryza sativa (Xiao et al., 2009; Almeida et al., 2017). The expressions of *BcTCP3*, *5*, *7*, *12*, *21*, *22*, *23*, and *24* displayed more than 30 fold up-regulation under ABA treatment. During drought treatment, *BcTCP2*, *3*, and *12* were up-regulated by more than 15 fold. *BcTCP3*, *12*, *21*, and *22* were also up-regulated by more than 30 fold under cold treatment. These results were consistent with previous research results in rice (Yang et al., 2013; Wang et al., 2014). Subcellular localization analysis demonstrated BcTCP3 and BcTCP21 were located in the nucleus, indicating they may function as transcription factors.

These results revealed that *BcTCP*s were associated with multiple abiotic stresses. Our study may be helpful for improving plant stress tolerance. This will provide a piece of vital evidence for detecting the molecular mechanisms of *TCP*s under abiotic stresses. The systematic characterization of *BcTCP*s in Pak-choi will provide a better foundation for further functional studies of this gene family in plant growth and development.

## Data Availability Statement

The original contributions presented in the study are included in the article/Supplementary Material, further inquiries can be directed to the corresponding author/s.

## Author Contributions

FH, CS, and YZ completed the experiments and wrote the manuscript. XH revised and approved the manuscript. All authors have read and agreed to the published version of the manuscript.

## Conflict of Interest

The authors declare that the research was conducted in the absence of any commercial or financial relationships that could be construed as a potential conflict of interest.

## Publisher’s Note

All claims expressed in this article are solely those of the authors and do not necessarily represent those of their affiliated organizations, or those of the publisher, the editors and the reviewers. Any product that may be evaluated in this article, or claim that may be made by its manufacturer, is not guaranteed or endorsed by the publisher.

## Footnotes

1
http://pfam.xfam.org/

2
https://web.expasy.org/protparam/

3
https://www.arabidopsis.org/

4
http://bioinformatics.psb.ugent.be/webtools/plantcare/html/

5
http://meme-suite.org/

6
http://wolfpsort.org/
